# Editorial: Insights in cellular neurophysiology: 2022

**DOI:** 10.3389/fncel.2023.1330849

**Published:** 2023-11-13

**Authors:** Lisa Mapelli, Jonathan Mapelli, Dieter Wicher

**Affiliations:** ^1^Department of Brain and Behavioral Sciences, University of Pavia, Pavia, Italy; ^2^Department of Biomedical, Metabolic and Neural Sciences, University of Modena and Reggio Emilia, Modena, Italy; ^3^Department of Evolutionary Neuroethology, Max Planck Institute for Chemical Ecology, Jena, Germany

**Keywords:** ferroptosis, GABA, olfactory sensory neurons, retinal ganglion cells, dendritic release, electromagnetic fields, daily two-photon imaging

This Research Topic invited Frontiers Editors to highlight significant challenges and recent accomplishments in the neuroscientific field and highlight future directions. Seven contributions were gathered, including original articles, a review, and a methods report.

Ferroptosis is a recently identified non-apoptotic and iron-dependent programmed cell death, characterized by intracellular iron ions accumulation. The involvement of ferroptosis in several disorders has been demonstrated, including neurodevelopmental disorders such as epilepsies. Giustizieri et al. reported that the ferroptosis inducer RSL3 causes persistent spontaneous interictal discharges in layer IV principal cells of adult mouse somatosensory cortex. These effects are similar to those obtained using several convulsant treatments, such as kainate or high potassium, and were associated with reduced GABAA-mediated inhibition. Therefore, the induction of ferroptosis in the cortical circuit altered the excitatory/inhibitory balance, which is well-known to be involved in several pathological conditions. The authors demonstrated the neuroprotective role of Vitamin E to rebalance redox homeostasis, which is compatible with the recognized role of reactive oxygen species in ferroptosis physiological induction.

Maintaining ion gradients is paramount for cell functioning, particularly for neuronal physiology. Many neuropathological conditions are driven by altered ion concentration in the intracellular or extracellular space, which might have several origins. The potassium-chloride cotransporter (KCC2) is a primary Cl^−^ extruder responsible for the low Cl^−^ concentration in the intracellular medium in mature neurons. As such, it is crucially involved in GABAA-mediated synaptic inhibition. Pethe et al. investigated the interaction between the KCC2 and the electrogenic sodium/bicarbonate cotransporter 1 (NBCe1), which is involved in pH regulation in neurons and glial cells. By combining several techniques, from immunoprecipitation to electrophysiology and imaging on hippocampal neurons, the authors described such interaction and provided a first characterization of its effect in the modulation of KCC2 activity.

Olfaction is a crucial sensory modality in many animals, including insects. Nevertheless, the intracellular machinery of the peripheral olfactory system is not entirely understood. Prelic et al. investigated the nitric oxide – cGMP pathway in the *Drosophila* antenna, combining electrophysiological and optical techniques with pharmacological manipulation. They first demonstrated the expression of the nitric oxide signaling machinery and tested the effects of activating and inhibiting the system at different levels. With a battery of tests, the authors reported no nitric oxide – cGMP pathway involvement in the *Drosophila* olfactory response. This evidence does not exclude that this pathway might take part in functions other than strictly the olfactory response and marked a further step in characterizing such a complex sensory modality.

The most crucial sensory modality in humans is undoubtedly vision. Neurodegenerative diseases characterized by retinal ganglion cell (RGC) death cause progressive and irreversible vision loss. RGCs are responsible for visual signal transduction and physiologically work under high exogenous stress levels, needing efficient neuroprotective systems to maintain homeostasis and proper functioning over time (Pietrucha-Dutczak et al., 2018). One of the main endogenous protective mechanisms relies on RNA-binding proteins, among which ELAVL/HuR has been reported to mediate the expression of most stress-related proteins in neurons. Pacwa et al. reported that *hur* silencing in rats decreased RGC function and viability *in vivo* during aging, showing that an adequate HuR protein level is crucial not only for survival but also for proper neurotransmission maintenance. The authors also provided evidence of HuR involvement in the pathological features of a glaucoma model. Furthermore, an interesting role of HuR pathways in mediating the efficacy of exogenous neuroprotective treatments is reported.

The secretory activity of neurons is crucial for the homeostatic regulation of several systemic mechanisms. Most neurosecretory neurons release both from axonal terminals and dendritic compartments. In this last case, are physiologically relevant stimuli enough to drive dendritic release or is there any contribution of purely electrical activity, like backpropagating action potentials (Ludwig and Leng, 2006)? In Korogod et al. the authors develop a multicompartmental computational model of magnocellular secreting neuron to evaluate the somato-dendritic transfer of electrical signals and its potential dependency on geometrical characteristics. The authors predict a crucial role for morphological features such as dendritic diameter, the number and size of dendritic varicosities or the volume of the peri-dendritic space defined by glial sheath wrapping. As a whole, the geometrical factors have a marked impact on electrical activity of these secretory neurons generating electrical decoupling between somas and dendritic compartments.

The interaction of electromagnetic fields with biological tissue has a long history that goes back to Galvani's (1791) classical experiments on frog legs to the fundamental work by Hodgkin and Huxley on nerve excitation (Hodgkin and Huxley, 1952). Sophisticated electrophysiological methods allowed then to identify ion channels as basis of tissue excitability (Verkhratsky et al., 2006). Another strain of development applied alternating electromagnetic fields on tissue and cell suspensions. Depending on the frequency, the electromagnetic field interacts with cell substructures such as surface charges, plasma membrane, proteins and may lead to resonance effects (Schwan, 1957). An application of the interaction between electromagnetic field and ion channels is reviewed by Abed et al. Here, the authors report the use of weak electromagnetic fields in the therapy of glioblastoms. Special attention is paid on effects of weak electrical fields on voltage sensors of ion channels and the authors discuss voltage-gated ion channels as possible targets of tumor electrotherapy.

The development of advanced techniques has often accompanied the neuroscientific breakthroughs of the last decades. Such improvements are opening unprecedented possibilities in terms of experimental design and reproducibly detailed information. Huang et al. presented an advanced method to investigate learning and long-term phenomena *in vivo* at the single neuron level, monitoring dynamic changes in individual dendritic spines in a population of neurons. The proposed technique combined daily two-photon calcium imaging during auditory associative learning, followed by targeted single-cell loose-patch clamp recordings, and electroporation of plasmid for enhanced chronic calcium imaging of dendritic spines in the targeted cell. This technique allows the analysis of behaviorally related cellular and subcellular dynamics and tracks long-term changes throughout behavior. The authors also commented on the possible promising combination with other cutting-edge techniques, such as two-photon optogenetics, and future improvements.

## Author contributions

LM: Writing—original draft, Writing—review & editing. JM: Writing—original draft, Writing—review & editing. DW: Writing—original draft, Writing—review & editing.
